# Understanding the Decision-Making Process Between Presenteeism and Absenteeism

**DOI:** 10.3389/fpsyg.2021.716925

**Published:** 2021-07-20

**Authors:** Daniela Lohaus, Wolfgang Habermann

**Affiliations:** ^1^Faculty of Social Sciences, Institute of Business Psychology, Darmstadt University of Applied Sciences, Darmstadt, Germany; ^2^H & L Karriereberatung, Lautertal, Germany

**Keywords:** sickness presenteeism, absenteeism, decision-making process, expectancy theory, experimental vignette study, motivation, attendance behavior

## Abstract

Due to their impact on various stakeholder groups, research on the global phenomena of sickness presenteeism (working despite illness) and sickness absenteeism (absence due to illness) is constantly growing. Most studies focus on identifying factors associated with the attendance behaviors. In contrast, there have been few theoretical approaches to explain the individual decision-making process for or against working while ill. Moreover, their empirical verification is still pending. In the present study, we refer to expectancy theory to theoretically explain how the decision is made. To empirically test the model predictions we applied experimental vignette methodology in an online survey with working adults. The hypotheses were confirmed in that the calculated and predicted decisions significantly matched the intentionally chosen decisions. The results contribute to a better theoretical understanding of the decision-making process and provide starting points for interventions to manage attendance behavior in organizations.

## Introduction

Absence from work because of illness (sickness absenteeism) and presence in spite of illness that would warrant absence from work (sickness presenteeism) have received considerable research attention (Ruhle et al., 2020). Many researchers view these phenomena as connected (e.g., Caverley et al., 2007; Bierla et al., 2013; Deery et al., 2014; Garrow, 2016) not only because of their high statistical correlation (Johns, 2010), but also because both attendance behaviors relate to the employees’ health (e.g., Demerouti et al., 2009; Hansen and Andersen, 2009; Janssens et al., 2013; Skagen and Collins, 2016). Further, they have a major economic impact for organizations due to reduced productivity (Collins et al., 2005; Iverson et al., 2010; Warren et al., 2011; Vanni et al., 2017) and disruption of work processes (Gosselin et al., 2013; Strömberg et al., 2017; Miraglia and Johns, 2021).

The majority of empirical studies has focused on the identification of correlates of the attendance phenomena (e.g., Johns, 2011; Miraglia and Johns, 2016, 2021) while little research has been done to understand the individual’s psychological processes leading to the decision to attend work or not in case of illness (Gosselin, 2018). Interestingly, sickness absenteeism and sickness presenteeism research has mainly developed along parallel paths although the phenomena are the result of a complex decision-making process that rules out the other alternative (Johns, 2010; Halbesleben et al., 2014; Lohaus and Habermann, 2019). Thus, scholars point to the imperative of a single theoretical framework that brings both concepts together (Johns, 2010, 2011; Halbesleben et al., 2014; Gosselin, 2018). In accord with this concern, a major aim of this paper is the theoretically founded explanation of the individual’s decision process between absenteeism and presenteeism.

### Research on the Decision Between Absenteeism and Presenteeism

Within both fields of research, there are theoretical approaches. Their focus lies mainly on factors influencing absenteeism and presenteeism and their effects (e.g., Nicholson, 1977; Aronsson and Gustafsson, 2005; Darr and Johns, 2008; Johns, 2010; Laaksonen et al., 2010; Lu et al., 2013; Miraglia and Johns, 2016, 2021; Zhou et al., 2016). The abundance of variables identified as relevant can be classified into four broad groups, namely factors related to the individual, the work, the organization, and the environment (Lohaus and Habermann, 2019). The theoretical frameworks mostly consider the attendance behaviors separately and do not explain how determinants interact at the point of decision between presence and absence in case of illness (Gosselin, 2018). They name relevant factors, but usually do not address the fact that attendance behaviors occur in contexts that are characterized by social dynamics (Johns, 2010) and thus, for each individual variables influencing the decision combine in a unique way. Therefore, to understand the decision between the mutual alternatives it is more promising to focus on the individuals’ psychological process of decision-making rather than the factors influencing the decision (Halbesleben et al., 2014). To our knowledge, there have been only very few approaches to study this attendance dynamic on the micro level (i.e., Halbesleben et al., 2014; Cooper and Lu, 2016).

The model by Cooper and Lu (2016) combines impact factors and psychological mechanisms. The authors draw on Bandura’s (1986, 2001) social cognitive theory to explain presenteeism. According to them “perceptions of self-efficacy and outcome expectations figure prominently intentions and goals of work involvement” (Cooper and Lu, 2016, p. 225). In addition to direct effects of self-efficacy and outcome expectations on presenteeism, they posit that intentions and goal systems formed on the basis of efficacy beliefs lead people to expect positive outcomes. These expectations lead to presenteeism, which in turn serves to attain performance. While this approach obviously focuses on the psychological mechanisms in the emergence of presenteeism, it does not address absenteeism, let alone the process how the individual reaches the decision between both attendance behaviors.

Halbesleben et al. (2014) apply dialectical theory (Baxter, 1990) to understand the relationship between employee and supervisor. Dialectical theory surmises that tensions or opposing forces affect social relationships. Dialectical tensions are assumed to emerge from three key contradictions: autonomy-connection, predictability-novelty, and openness-closedness, with the latter relating to power due to the sharing of information. The authors transfer this approach to the work setting and postulate that the decision to attend work or not in case of illness is a means to manage experienced tensions on the side of the employee. These tensions are presumed to result from differing expectations of supervisor and employee. The decision for absenteeism or presenteeism “is a reflection of one’s desire to be more or less involved in a relationship with his or her supervisor” (Halbesleben et al., 2014, p. 178). The authors derive different strategies subordinates might employ to deal with these tensions, such as denying that the contradiction exists or compromising between the two poles of a contradiction. The choice of strategy is based on the subordinate’s and the supervisor’s respective location on a particular continuum. Depending on which strategies employees choose with regard to the various contradictions, either presenteeism or absenteeism will result. The merits of the paper are undeniably to bring both attendance behaviors under one theoretical umbrella and to focus on the motivation that drives the behavior. However, although supervisors have proven to be an influence factor (e.g., Nyberg et al., 2008; Nielsen and Daniels, 2016; Dietz and Scheel, 2017; Schmid et al., 2017), we see a major shortcoming in its restriction to the supervisor-subordinate-dyad. Due to this limitation, the authors focus on a small part of the work-related factors and leave aside other work-related influences as well as factors relating to the person, the organization, and the environment. Thus, it remains unclear how the decision between absenteeism and presenteeism can be explained independently of the supervisor-subordinate-dyad. Further, the model has yet to be tested empirically.

### Aims of the Study

Thus, although acknowledging that attendance behavior is “to some extent intentional … and grounded in a goal-directed decision process” (Karanika-Murray and Biron, 2020, p. 246) we still do not understand the role motivation plays and the psychological factors driving the decision (Knani et al., 2018). In accord with this concern, this paper has two objectives. First, it demonstrates that one can draw on an established theory for work settings, i.e., Vroom’s (1964, 1995, 2005) expectancy theory of work motivation, to explain theoretically the choice of attendance behavior on the micro level. Second, we show empirically that Vroom’s theory is appropriate to predict the decision process of employees in an experimental setting. For this, we apply experimental vignette methodology and two different statistical approaches to analyze the data. Benefits of this research are its contribution to theory building thereby unifying absenteeism and presenteeism under one roof to gain the holistic view Ruhle et al. (2020) call for. The better understanding of the decision-making process might enable effective managerial interventions to support and promote occupational health, employee performance, work organization, and organizational productivity.

To achieve the first objective, we begin by describing the basic ideas of Vroom’s theory before applying them to attendance behavior. After deriving the hypotheses, the study design is presented with the development of the material. The empirical part serves to achieve the other goal, i.e., to show that the theory is applicable in principle to explain the decision-making process.

### Vroom’s Expectancy Theory

Scholars agree that although research has established correlates of attendance behavior “the personal account of “why” still needs to be systematically explored, namely, what consequences do people expect for not/coming to work when ill” (Cooper and Lu, 2016, p. 224). It is not yet clear how employees actually reach the decision and especially how they make the compromises between health and motivation to work (Knani et al., 2018; Karanika-Murray and Biron, 2020). Gosselin (2018) stresses the fact that the individual decision process is unique. Also Karanika-Murray and Biron (2020, p. 246) highlight the fact that because employees “will differ in the purpose, functions, and goals that presenteeism serves for them, they will also differ in the ways that their health and performance are further affected as a result of enacting presenteeism.”

To investigate a motivationally driven individual decision process in work settings it is obvious to apply the expectancy theory of work motivation (Vroom, 1964, 1995, 2005). It has been rated as one of the most important and scientifically valid theories of organizational behavior (Miner, 2003) and as applicable to diverse settings (Pinder, 2016). In the following, we describe the central assumptions of Vroom’s expectancy theory.

The basic tenet of the theory is that the motivational force (MF) behind the intention to achieve a specific goal is the mathematical product of expectancy (E), instrumentality (I), and valence (V; Vroom, 1964, 1995). Because of these main components, the approach has been termed “valence-instrumentality-expectancy theory”; in short “VIE” theory (Pinder, 2016, p. 363; Vroom, 2005, p. 254). The three components are conceptualized as perceptions and beliefs of the individuals that stimulate and direct their behavior. Expectancy involves an action-outcome link, while instrumentality is an outcome-outcome-association (Vroom, 1964, 1995). Pinder gets to the heart of the theory when describing expectancy, instrumentality, and valence in the following way (Pinder, 2016, p. 364):

‘More specifically, VIE theory proposes that behavior is instigated and directed to the extent that:
(1) people believe that the behavior will lead to outcomes such as job performance;
(2) people believe that such outcomes will be rewarded; and
(3) people value those rewards.”

Valence is a preference for a desired outcome (or reward) among various outcomes that represents the person’s anticipated value of or satisfaction with achieving this outcome. Vroom posits that people pursue several desired outcomes at a time and their behavior is a result of conscious and rational choices between alternative behaviors due to the maximal motivational force behind the alternative behaviors. These preferences are also termed goals (e.g., Pinder, 2016) or utility judgments, reflecting the attractiveness of the outcomes (Seo et al., 2004; Vroom, 2005). They can be held among different types of outcomes (such as social interactions, monetary rewards, promotion, job security) or different levels of particular outcomes (e.g., a preference for a higher rate of pay as compared to a lower rate of pay, having more leisure time as compared to less). Valences of outcomes are related to the individuals’ relatively stable dispositions, i.e., needs and motives (Vroom, 2005).

Instrumentality is a probability belief linking one outcome to another (Pinder, 2016). It represents the subjective perception of how outcomes of individuals’ actions are related to their goals and it can be positive or negative. Thus, it asserts the instrumental “power” in attaining a certain goal and satisfying a motive (Vroom, 2005). For example, working overtime holds positive instrumentality for obtaining a promotion while it holds negative instrumentality for spending time with one’s family.

Expectancy refers to the individuals’ subjective probability, i.e., their degree of certainty to which they assume that a specific action or effort will lead to a certain performance or outcome (result). It depends on the individuals’ self-efficacy (Bandura, 1977), i.e., the belief in their capabilities. Expectancy thus depends to a certain extend on the experiences of individuals in their private and work settings.

When it comes to a decision, the essence of the VIE model means that an individual selects from various action options the one(s) with the strongest positive or weakest negative motivational force. Vroom (1964, 1995) elaborated his theory specifically on the goals of occupational choice, job satisfaction, and performance. Since then is has been applied to a variety of settings, such as motivation to take on specific work roles (Barba-Sánchez and Atienza-Sahuquillo, 2017), job satisfaction (Davidescu and Eid, 2017), performance-related behavior (Puplampu and Adomako, 2014; Shweiki et al., 2015) and its perception (Wardayati, 2016), and pro-environmental behavior (Baumhof et al., 2017; Kiatkawsin and Han, 2017). However, to our knowledge, it has not yet been applied to the decision process between presenteeism and absenteeism.

### Adapting Vroom’s Expectancy Theory to the Context of Absenteeism and Presenteeism

In accord with the first aim of the paper, we transfer Vroom’s (1964, 1995) expectancy theory to the context of attendance behavior to understand the individual’s decision-making process between presenteeism and absenteeism. We propose that this decision can be explained in the following way: When employees are sick, the question for them is whether to call in sick or work despite illness. These are the two options for action in this specific situation. According to expectancy theory, the choice between the two options depends on which one has greater motivational potential. This motivation potential in turn depends on the probability with which the individuals believe they will be able to achieve their goals by taking one or the other course of action. This assumption is consistent with our knowledge that employees choose attendance behavior with respect to satisfying a number of goals they value (Karanika-Murray and Biron, 2020). However, they cannot attain these goals directly and often solely by their own means, since circumstances and other persons’ behaviors normally do come into play. Therefore, they have to strive for intermediate outcomes that they can influence and that they believe to be instrumental for achieving their goals. Further, they must decide whether presenteeism or absenteeism has a greater likelihood of leading to these intermediate outcomes.

In terms of expectancy theory that means, when employees who are scheduled for work realizes that they are in a medical condition that justifies calling in sick, they will make a conscious decision. They will think about relevant goals in this situation and how highly they value these goals (valences). They will speculate on which outcome is instrumental or detrimental for reaching these goals (instrumentality). Finally, they will reflect on how presenteeism and absenteeism might affect these outcomes (expectancy). They will choose that attendance behavior that – in sum – seems the best trade-off for attaining their goals (motivational force).

We illustrate this with an example. Imagine employees would very much like (valence) to be accepted and feel comfortable in the work team (goal). To attain their goal, they might believe it makes sense (expectancy) to complete their work tasks and thus avoid extra work for their colleagues (result). Further, they might be convinced that it is expedient (expectancy) to protect all employees’ health (result). In the first case, they might reason that presenteeism increases the likelihood of avoiding extra work for the other team members, whereas absenteeism seems preferable in order to avoid spreading germs and thus transmitting infection to them. Of course, employees usually do not pursue just one goal, but several at the same time, which may even contradict each other. For example, another goal of the employees that they value highly (valence) could be to stay healthy to ensure their employability. If they choose presenteeism to accomplish their tasks and avoid extra work for their team members (result), they would not be able to recover (result), which would be detrimental (instrumentality) to obtaining good health (goal). Absenteeism would surely (expectancy) give them time to rest and recover (result), furthering (instrumentality) their goal of ensuring good health, but would imply (expectancy) that their colleagues have to fill in for them (result), which could be harmful (instrumentality) for their goal of being an accepted team member.

We apply Vroom’s propositions to the context of attendance behavior in case of sickness in the following way: The actions employees have to choose between are presenteeism (p) and absenteeism (a). The employees have i goals (outcomes) that they value in this situation, and they consider j results that should further goal attainment. The decision in favor of presenteeism or absenteeism will depend on which action has a greater total amount of motivational force behind it. The motivational force of an action with regard to one result and one goal comes to the mathematical product of the valence of the goal weighted with the subjective probability that the result will be instrumental for it (instrumentality) and the probability with which the taken action will lead to the result (expectancy). In view of several results and goals that normally would be considered, the total amount of motivational force behind an action is the sum of the possible products of these factors. In mathematical terms the eq. 1:

(1) M⁢F=E*I*V

has to be split into the eqs. 2 and 3:

(2) MF=pΣi(ΣEj*jpIi,j*Vi)

(3) MF=aΣi(ΣEj*jaIi,j*Vi)

with MF^*p*^ = motivational force behind action option p (presenteeism), MF^*a*^ = motivational force behind action option a (absenteeism), E^*p*^_*j*_ = expectancy of action p prompting result j, E^*a*^_*j*_ = expectancy of action a prompting result j, I_*i*_,_*j*_ = instrumentality of result j with regard to goal i, V_*i*_ = valence of goal i. The comparison between the two motivational forces determines the decision in favor of presenteeism or absenteeism: If MF^*p*^ is greater than MF^*a*^ the employees will decide for presenteeism, if it MF^*a*^ exceeds MF^*p*^ they will chose absenteeism.

**FIGURE 1:**
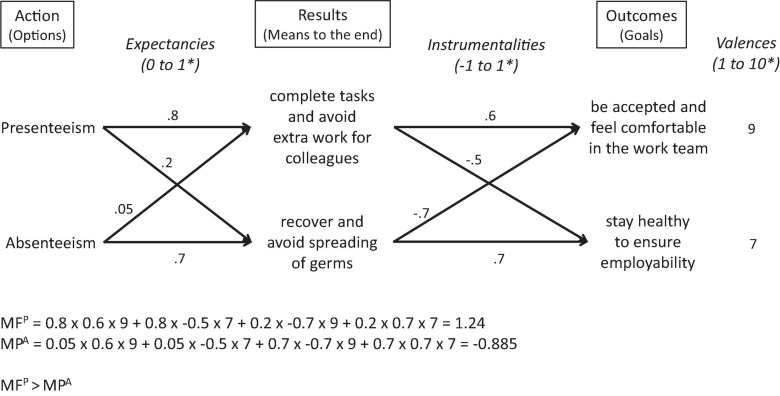
Example of the application of expectancy theory to attendance behavior. *Higher values represent higher subjective probabilities and valences.

Figure 1 illustrates this relationship for the above example. The values for expectations, instrumentalities and valences are plausible but arbitrarily chosen.

In pursuit of the second aim of the paper, we want to show empirically that expectancy theory is applicable to the decision-making process in case of illness. To test the applicability of the model, we proceed as follows. We create a study context comprising vignettes in which participants are requested to imagine that they wake up in the morning when scheduled for work and realize that they are sick (Gosselin, 2018). We ask them to rate from their point of view valences of goals, instrumentalities, and expectancies in this situation, and finally let them decide whether they would work in spite of sickness or not. These ratings are entered into the abovementioned formulas in order to calculate the motivational forces and determine the accordingly expected decision. To verify the applicability of expectancy theory, we compare the decision chosen by participants with the one calculated. We posit that the VIE calculus represents the considerations underlying the decision between presenteeism and absenteeism and therefore hypothesize:

Hypothesis 1: The degree of correspondence between the decision consciously chosen by the individual and the calculated decision will be significantly above chance level, which is 50%.

Since in this study design the dependent variable is dichotomous (working in spite of sickness or not) it is obvious to apply binary logistic regression analysis (Field, 2018) with the independent variables valence, instrumentality, and expectancy. In applying binary logistic regression, maximizing the log-likelihood value yields the best fit between participants’ discretionary decision and the probability that their assessment of the VIE factors will result in presenteeism or absenteeism. In testing the applicability of the VIE theory, we are interested in the goodness of prediction, i.e., the model fit, as it represents the process of decision-making. In assessing the model fit as a whole, the focus is on how well the independent variables in sum contribute to the separation of the dichotomous response alternatives. Our statistical analysis does not focus on the relative influence of the independent variables, which represent content factors that vary by individual and context. Therefore, we derive the following hypothesis to show the applicability of the VIE model:

Hypothesis 2: The variables of the VIE model explain statistically significantly the decisions between presenteeism and absenteeism.

## Materials and Methods

**FIGURE 2:**
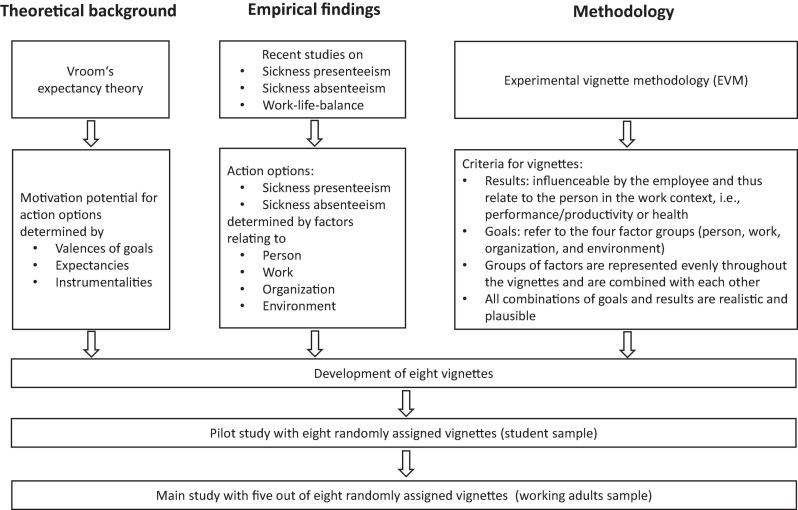
Overview of study.

In this section, we describe the development of the study design and material, the procedure, and the data analysis. Figure 2 gives an overview of the study and depicts how the method of constructing the material relates to the theoretical background and to empirical findings.

### Study Design, Development, and Test of Vignettes

Scenarios have previously been used to study attendance behavior, specifically reactions to absenteeism (e.g., Patton, 2011; Addae et al., 2013). We applied this approach following the experimental vignette methodology (EVM) since “EVM allows researchers to include factors that are relevant to the research question while excluding those that might confound the results” (Aguinis and Bradley, 2014, p. 357). A vignette is “a short, carefully constructed description of a person, object, or situation, representing a systematic combination of characteristics” (Atzmüller and Steiner, 2010, p. 128). In the construction of the scenarios we closely followed the recommendations provided by the authors (Atzmüller and Steiner, 2010; Aguinis and Bradley, 2014).

**FIGURE 3:**
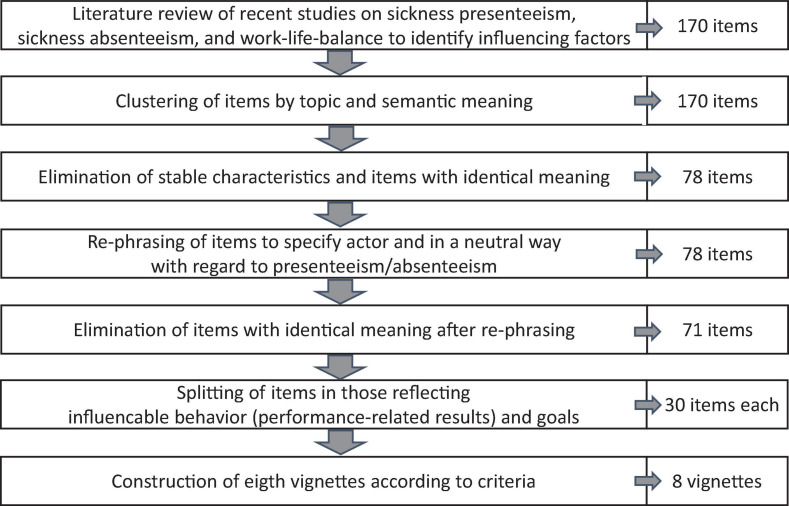
Development of vignettes.

**FIGURE 4:**
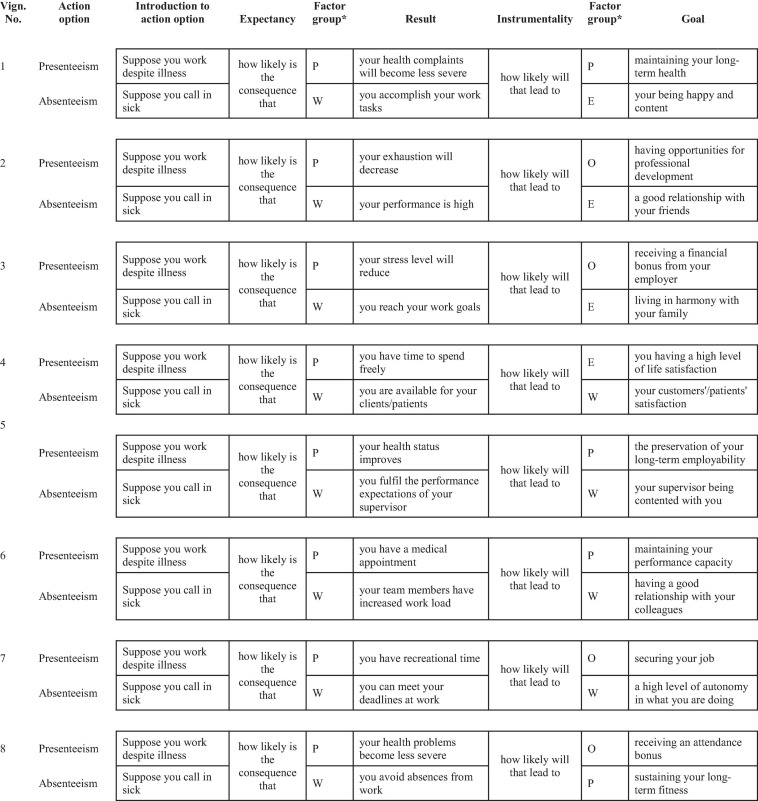
Vignettes used in the empirical study (translated by the authors). *P = Person, W = Work, O = Organization, E = Environment; not shown in the questionnaire.

We developed written vignettes in the following steps (see Figure 3). First, we wanted to capture aspects broadly covering the four groups of relevant factors as stated in the most comprehensive content model of presenteeism (Lohaus and Habermann, 2019), which are factors related to the individual, the work, the organization, and the environment. Thus, we performed a review of recent empirical studies and reviews on the topics of presenteeism (e.g., Miraglia and Johns, 2016; Knani et al., 2018; Lohaus and Habermann, 2019), absenteeism (e.g., Biron and Bamberger, 2012; Addae et al., 2013; Rostad et al., 2015; Pichler and Ziebarth, 2017), and work-life-balance (e.g., Nilsen et al., 2017; Sirgy and Lee, 2018). That search resulted in 170 items. We clustered them by topic (e.g., health, performance, reward system) and eliminated semantically redundant items. Further, we removed stable characteristics of employees that are not applicable to phrase results and goals (e.g., conscientiousness), leaving a pool of 78 items. We phrased items in a way that makes clear who is the actor (e.g., instead of “risk of higher error rate” → “the risk increases that you make errors”) and that they were not associated with either absenteeism or presenteeism (e.g., instead of “it is good for you to work” → “it is good for you to behave in this way”). This resulted in a further reduction of items with similar meanings. Then, to limit complexity of the vignettes, we constructed them to consist of the minimum of two results and two goals. Each vignette was phrased according to the following criteria: (1) In accord with expectancy theory, results must be influenceable by the acting person (the employee) and thus relate to the person in the work context, i.e., performance/productivity or health, while goals refer to the four factor groups (person, work, organization, and environment). (2) The four groups of factors are represented evenly throughout the vignettes and are combined with each other. (3) All combinations of goals and results are realistic and plausible. This resulted in eight vignettes. Figure 4 depicts the vignettes in separate boxes, each comprising of the two alternative action options (presenteeism and absenteeism) with their respective introductory texts, and the question introducing the assessment of the likelihood of achieving two different results when deciding for presenteeism or absenteeism. Further, you find the introductory question for assessing the instrumentality with which the results will bring forth the two given goals. Directly behind both questions, the factor groups are denoted that the given results and goals belong to.

**FIGURE 5:**
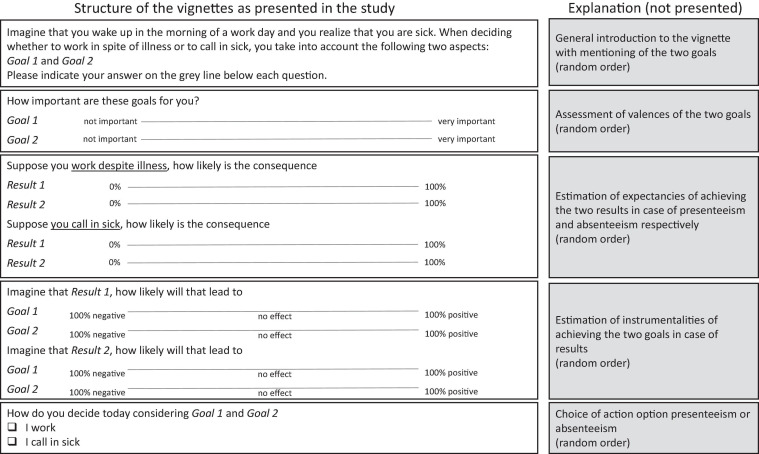
Structure of vignettes as presented (translated by the authors) and explanation of the structure.

Following the suggestion of Gosselin (2018), each vignette started with the identical phrase: “Imagine that you wake up in the morning of a work day and you realize that you are sick. When deciding whether to work in spite of illness or to call in sick, you take into account the following two aspects” (followed by the two goals of the vignette). Then, participants were required to assess the importance of these goals for them in this situation (valences). Next, they rated the probabilities of performing both presented results when deciding for working in spite of illness or calling in sick (expectancy). Further, they were asked to assume that these results occurred and how probable their occurrence would affect their goals (instrumentality). Finally, they had to decide whether to work or call in sick in this situation (decision). Within each group of variables (valences, expectancies, instrumentalities, and decision), items were presented in random order. With the exception of the decision, which was dichotomous, we used sliders for the ratings with their endpoints labeled. Valences ranged from “not important” (1) to “very important” (10); expectancies from “0%” to “100%,” and instrumentalities from “100% negatively” to “100% positively” with the scale midpoint labeled “no effect” (see Figure 5).

A pilot study with working students showed that all eight vignettes worked; however, processing time for the entire set was very high and led to dropouts. Hence, for the main study, we decided that each participant would receive a random set of five out of the eight vignettes (Atzmüller and Steiner, 2010). Although we still expected a long processing time, this procedure was deliberately chosen because it ensured that we incorporated all factor groups in the study that had previously been proven to be relevant.

### Procedure

Working adults were invited to the study via social media. As an incentive for participation, the researchers pledged to donate one Euro to a charitable organization for each completed survey. Before starting the survey, we informed participants about the goals of the study and ensured them that they could withdraw their participation from the anonymous survey at any time without incurring any negative consequences. We let them know that they would be asked to give their opinions, and that their data would be collected for scientific purposes only and stored for 10 years. After the participants provided their written informed consent to participate in this study, the survey started. The general instruction informed participants that they would be presented with five different situations, which they should try to image as vividly as possible, and they should answer all questions spontaneously according to their personal opinion. After completing the five vignettes, participants received items concerning their past attendance behavior: (a) how many days during the previous 12 months they had worked despite feeling sick and having a justification for staying home (e.g., Aronsson and Gustafsson, 2005; Pohling et al., 2016) and (b) how many days they had stayed home due to sickness (e.g., Gerich, 2016). The final questions referred to demographic information. The median time for completion was 13.2 min.

### Data Analysis

Data analysis was performed using IBM SPSS version 27.0 (IBM SPSS Inc., Chicago, IL, United States). The level of statistical significance was set at *p* < 0.05 in all the analyses conducted.

Frequencies, ranges, means, and standard deviations were determined to describe the sample. We recorded presenteeism days (i.e., presenteeism rate or frequency) as they depend on the number of health events and are an indicator of health status or vulnerability to illness (Gerich, 2016). On this basis, we calculated the sickness presenteeism prevalence as the percentage of participants having shown presenteeism during the 12 month before the survey. Further, sick days were determined as the sum of presenteeism and absenteeism days (Gerich, 2016; Lohaus and Röser, 2019). Sickness presenteeism propensity, which reflects an individual’s probability of opting for sickness presence rather than sickness absence in the case of illness (Gerich, 2016), was computed as presenteeism frequency divided by the number of sick days (Biron et al., 2006; Gerich, 2016; Lohaus and Röser, 2019). Thus, it offers information with regard to the decision-making process of the individual.

To test the first hypothesis that the correspondence between chosen decisions and the decisions calculated in accordance with the VIE model is significantly above chance level, we used a *t*-test. We applied binary logistic regression analysis to test the second hypothesis. Linearity was tested assessed using the Box-Tidwell (Box and Tidwell, 1962) procedure. Bonferroni-correction was applied to all ten terms in the model (Tabachnick and Fidell, 2018). All variables were found to follow a linear relationship. Correlations between predictor variables were low (*r* < 0.70), indicating that multicollinearity was not a confounding factor in the analysis (Schroeder, 1990; Tabachnick and Fidell, 2018). Studentized residuals, leverage values, and Cook distances were considered to identify outliers. No case was consistently identified as an outlier, so all values were included in the analysis (Field, 2018). To test hypothesis 2, we classified chosen versus predicted responses and used *chi*-square with pseudo *R*-square (Nagelkerke) and Cohen’s *f*-square as indicators of effect size. Goodness-of-fit was assessed using the Hosmer-Lemeshow-Test. Since every vignette comprised different factors (independent variables), we performed the analysis for each vignette separately to test the fit of the model.

## Results

The results section consists of three parts. First, we report the sample characteristics with their demographics, their health data, and their attendance behavior in the 12 months preceding the study. Then we list the descriptive results of the vignettes before reporting the results of the statistical tests of the hypotheses.

### Descriptive Analysis of the Sample

Of 294 people who started the survey, 202 completed it (68.7%). Seven participants were excluded from the analysis due to their employment status (i.e., volunteer worker, other). The remaining sample of 195 participants consisted of 160 employees, 9 self-employed people, 16 civil servants, and 10 trainees. One hundred fifteen participants (59%) were women, and 79 (41%) were men; one person did not indicate the gender. Participants’ ages ranged from 19 to 65 years with a mean of 43 years (*SD* = 12.0). The mean amount of work experience was 19.8 years (*SD* = 12.1) with a minimum of one and a maximum of 44 years. One hundred forty-five participants (74.4%) reported working full time, 78 (40.0%) had a supervisory position. 26.7% of participants worked in financial, IT, and business services, 22.6% in the industry, 13.8% in civil service and administration, and 12.8% in education, research, and culture. A university degree was held by 70.3% and 13.8% indicated vocational training as their highest educational qualification. Descriptive information about the attendance behavior of the sample during the 12 months preceding the survey is given in Table 1.

**TABLE 1:** Characteristics of the study group with regard to attendance behavior.

**Data base**	**N**	**Presenteeism rate (days) *M* ± *SD***	**Absenteeism rate (days) *M* ± *SD***	**Sick days *M* ± *SD***	**Presenteeism prevalence (%)**	Presenteeism propensity** *M* ± *SD*
Complete sample*						
Including long-term sick participants	179	12.0 ± 38.0	5.9 ± 14.3	17.9 ± 40.7	74.9	
Excluding long-term sick participants	170	4.3 ± 5.0	4.8 ± 5.6	9.2 ± 8.3	74.1	
Subsample of participants reporting sick days						
Including long-term sick participants	162	13.2 ± 39.7	6.6 ± 14.9	19.8 ± 42.3	84.4	0.49 ± 0.35
Excluding long-term sick participants	153	4.8 ± 5.1	5.4 ± 5.6	10.2 ± 8.2	82.4	0.47 ± 0.34

*16 participants did not report their attendance behavior and thus were not included in this analysis. **Presenteeism propensity can only be calculated for participants with sickness days > 0 (lower part of the Table).

### Descriptive Analysis of Vignettes

Table 2 lists the descriptive results for each vignette. On average, each vignette was rated by 122 participants, with a range of 114 to 132. The different number of participants per vignette results from the random drawing of five out of eight vignettes. The mean percentage of chosen decisions for presenteeism was 32.1 (range: 25.0 to 40.9) and for calculated decisions was 28.3 (range: 15.1 to 57.6). The mean percentage of chosen decisions for absenteeism was 67.9 (range: 59.1 to 75.0) and 71.7 (range: 42.4 to 84.9) for calculated decisions. The agreement of chosen and calculated decisions across vignettes ranged from 53% (vignette 7) to 75.4% (vignette 8) with a mean of 65.4%.

**TABLE 2:** Descriptive analyses of the vignettes.

		**Decision for presenteeism (in %)**		**Decision for absenteeism (in %)**		**Consistency of chosen and calculated decision (in %)**
**Vignette**	***N***	**Chosen**	**Calculated**	**Chosen**	**Calculated**	
1	127	30.7	25.2	69.3	74.8	64.6
2	124	25.0	18.5	75.0	81.5	67.7
3	117	31.6	19.7	68.4	80.3	74.4
4	114	37.7	46.5	62.3	53.5	54.4
5	115	31.3	23.5	68.7	76.5	69.6
6	120	30.8	19.2	69.2	80.8	65.0
7	132	40.9	57.6	59.1	42.4	53.0
8	126	28.6	15.1	71.4	84.9	75.4
Average	122	32.1	28.3	67.9	71.7	65.4

### Hypothesis Testing

To test hypothesis 1, participants’ ratings of the variables (valences, instrumentalities, and expectancies) were processed for each vignette according to eqs. 2 and 3 in order to determine which decision individuals should have made according to the VIE calculus (calculated decision). Then we computed the percentage of matches between the calculated decision and the respective decision consciously chosen by the participants (chosen decision) across the five vignettes they rated. Results supported hypothesis 1: They showed an average match of 65.4% between the calculated decision and the chosen decision. This result was significantly different from chance and in the expected direction, *t*(194) = 8.93, *p* < 0.001. The effect size was Cohen’s *d* = 0.64, which represents a medium to large effect (Cohen, 1988).

To test hypothesis 2, we performed a binary logistic regression analysis for each vignette. Results are displayed in Tables 3, 4. For six of eight vignettes the binary logistic regression model was statistically significant, i.e., the variable model was significantly better than the null model. Improvements by using the variable model compared to the null model ranged from 18% to 44% with an average of 29% (Nagelkerke). Effect sizes calculated as Cohen’s *f*^2^ ranged from 0.22 to 0.77 with an average of 0.43, which represent a strong effect (Cohen, 1988). Goodness-of-fit assessments indicated a good model fit for six of eight vignettes. The classification of chosen versus predicted decisions as shown in Table 4 pictures these calculations. Overall percentage of accuracy in classification was 75.2%. Thus, results supported hypothesis 2.

**TABLE 3:** Fit of the variable model as compared to the zero-model (Omnibus-test) for each vignette and indicators of significance and goodness of fit (binary logistic regression).

	**Omnibus test**			**Effect size**		**Goodness of fit**		
**Vignette No.**	***χ*^2^**	**df**	***p***	Pseudo R^2^*	**Cohen’s *f*^2^**	***χ*^ 2^**	**df**	***p***
1	38.49	10	0.000	0.37	0.58	9.17	8	0.328
2	16.69	10	0.081	0.19	0.23	2.55	8	0.960
3	24.95	10	0.005	0.27	0.37	20.78	8	0.008
4	23.10	10	0.010	0.25	0.33	10.07	8	0.260
5	36.76	10	0.000	0.38	0.62	5.85	8	0.664
6	16.69	10	0.082	0.18	0.22	8.09	8	0.425
7	26.81	10	0.003	0.25	0.33	16.62	8	0.034
8	45.73	10	0.000	0.44	0.77	11.79	8	0.161
Average				0.29	0.43			

*Nagelkerke.

**TABLE 4:** Level of correspondence of null model and variable model (binary logistic regression).

	**Correspondence (%)**	
**Vignette No.**	**Null model**	**Variable model**
1	69.3	78.0
2	75.0	79.0
3	68.4	77.8
4	62.3	71.9
5	68.7	79.1
6	69.2	70.8
7	59.1	68.9
8	71.4	82.5
Average	67.6	75.2

## Discussion

Sickness presenteeism and sickness absenteeism are global phenomena with a high prevalence rate, and they have been stimulating an ever increasing amount of research. While absenteeism has a long research tradition, the study of presenteeism has only gained momentum in the last two decades. Two aspects stand out when reviewing previous research: First, only a minority of studies have examined absenteeism and presenteeism together. Second, they have focused on identifying antecedents and consequences, so comprehensive content models of relevant factors now exist (e.g., Johns, 2010; Miraglia and Johns, 2016, 2021; Lohaus and Habermann, 2019), but individual decision-making has largely been ignored. This study addresses this gap and clarifies the process of decision-making in order to provide a more holistic understanding of the behavior (Ruhle et al., 2020).

Specifically, the paper had two objectives, both of which were achieved. First, we explained how the individual’s decision to work or not in case of illness can be pictured by Vroom’s expectancy theory (Vroom, 1964, 1995). Second, we demonstrated empirically that this approach is able to predict the decision process in an experimental setting. The findings are discussed below.

In summary, the results of the paper show that the application of Vroom’s expectancy theory to the decision between sickness presenteeism and sickness absenteeism offers a promising approach to explaining how the decision in question in principle comes forth. Vroom’s mathematical calculation scheme predicts the discretionary outcome of the decision-making process with significant strength. Furthermore using binary logistic regression analysis demonstrates that the variables derived from Vroom’s expectancy theory are also beyond the mathematical calculation a very good predictor for the chosen action in case of sickness.

### Theoretical Contribution

We have identified only one approach that attempts to explain the decision process between absenteeism and presenteeism at the micro level. Halbesleben et al. (2014) refer to dialectical theory to infer the individual’s choice. Although they have provided the most detailed explanation to date, they restrict it to the supervisor-employee dyad and do not consider other influencing factors. Furthermore, the authors have limited themselves to the theoretical derivation and have not yet empirically tested their assumptions.

To remove these limitations and to extend our knowledge with regard to an employee’s decision-making process when ill, with referring to Vroom’s expectancy theory, we drew on a more general theoretical approach and tested its assumptions empirically.

First, with regard to the aim to refer to a broader theoretical approach, the application of Vroom’s expectancy theory is useful for several reasons: It is a highly recognized theory of motivation for the work context (Miner, 2003), that is continuously applied to study decision-making processes. It has been supported by research in which it was used to make correct predictions of subjectively relevant decisions (e.g., Puplampu and Adomako, 2014; Shweiki et al., 2015; Wardayati, 2016; Barba-Sánchez and Atienza-Sahuquillo, 2017; Davidescu and Eid, 2017). It assumes that personally relevant goals and the subjective assessment of their attainability significantly affect the motivation to act. Thus, it adequately reflects the understanding that attendance behavior is a motivationally driven and goal-directed decision (Steers and Rhodes, 1978; Knani et al., 2018; Karanika-Murray and Biron, 2020). As urged by various researchers (e.g., Halbesleben et al., 2014), it unifies the decision to work or not to work in the event of illness under a common theoretical umbrella. It enables the simultaneous consideration of presenteeism and absenteeism, which are linked by a single decision. Vroom’s expectancy theory belongs to the process theories (Steers et al., 2004) and therefore allows describing the weighing of behavioral alternatives without reference to specific goals and influencing factors. Since our approach is not limited to the dyadic system of supervisor and subordinate, it extends the explanation of Halbesleben et al. (2014).

Second, the results of the empirical study supported both hypotheses. The correspondence of the participants’ intentionally chosen decisions with the decisions calculated according to the formulas derived from Vroom’s expectancy theory was above chance level. It thus demonstrated the latter’s applicability in principle. Additional statistical support was gained by employing binary logistical regression analysis. For the majority of the settings tested (vignettes), the statistics using Vroom’s expectancy theory variables significantly predicted the choices made and, on average, had medium (Nagelkerke) and strong (Cohen) effect sizes. The successful empirical testing of the theory’s applicability to attendance behavior expands our knowledge relative to previous approaches (Halbesleben et al., 2014).

### Managerial Implications

“In the contemporary employment-at-will context, employees make a voluntary decision to attend work prior to each working shift.” (Halbesleben et al., 2014, p. 189) and this decision is based on a subjective evaluation of their own health status (Johns, 2010; Karanika-Murray and Cooper, 2018). Thus, understanding the individual’s decision-making process when choosing between sickness presenteeism and sickness absenteeism is essential, both for the advancement of theory building and for the attendance management in organizations. So far, studies on attendance behavior have only examined a few influencing factors or correlates at a time. In reality, however, a large number of factors that are highly individual always play a role (e.g., Karanika-Murray et al., 2021). These aspects can influence each other and can be contradictory to each other. Vroom’s theory takes into account precisely this interaction of factors and their weighing by the individual. As a process theory, it thus offers a framework in which the relevant factors for the individual decision are brought together.

Of course, practitioners responsible for attendance management in organizations, such as HR managers, organizational health managers, and supervisors, cannot change their employees’ goals and their importance to them. However, the knowledge of how employees make the decision helps organizational stakeholders control this behavior to mitigate negative economic impacts and health consequences, as well as disruptive effects on work organization. They can influence the instrumentalities, i.e., the links between the behavioral results of their employees and the likelihood that those results will lead to the desired goals. This provides a valuable starting point for actively managing attendance behavior. Research has identified a variety of factors related to attendance behaviors that are under the control of employers. To name just a few, the importance of social support (Saijo et al., 2017; Nielsen et al., 2019; Yang et al., 2019; Aronsson et al., 2020), attendance cultures or climate (Thun et al., 2013; Løset et al., 2018; Mach et al., 2018; Martinez et al., 2018; Ferreira et al., 2019; Ruhle and Süß, 2019), reward systems (Della Torre et al., 2015; Rostad et al., 2017), and working conditions (Gerich, 2014; Jourdain and Vézina, 2014; Yang et al., 2016; Ferreira, 2018) should be noted here. For example, it is reasonable to assume that employees will stay home in the event of illness if they know that their replacement is well arranged (Miraglia and Johns, 2016) and they do not have to fear that their absence will incur the anger of their colleagues. This should apply at least if no other relevant goals of theirs override these considerations.

Consideration of individual goals and their value to the individual also fits well with recently published literature that attendance behavior is used to achieve positive effects (e.g., Demerouti et al., 2009; Giæver et al., 2016; Van den Broeck et al., 2016; Whysall et al., 2018; Gerich, 2020; Karanika-Murray and Biron, 2020; Lohaus et al., 2021). This is noteworthy in that most studies addressing the consequences of presenteeism refer to its negative effects on the individuals’ health (e.g., Bergström et al., 2009; Taloyan et al., 2012; Conway et al., 2014; Skagen and Collins, 2016), work performance and ability (e.g., Gustafsson and Marklund, 2011; Chen et al., 2021), or work attitudes (e.g., Karanika-Murray et al., 2015).

In the aftermath of the Covid-19 pandemic it may be expected that individuals’ goals pertaining to the protection of their health gain in importance relative to work-related goals. Whether this may lead to increased absenteeism depends on the individuals’ mind set. People who perceive working while ill will have a positive impact on their health and well-being (e.g., Rosso et al., 2010; Van den Broeck et al., 2016; Miraglia and Johns, 2018) will probably exhibit more presenteeism, while those who believe their health will benefit from rest will presumably opt more often for absenteeism.

### Strengths, Limitations, and Future Directions

A strength of the study is that it not only theoretically explains the individual decision process between sickness presenteeism and sickness absenteeism, but also empirically tests the applicability of the explanatory model. This study used thoroughly developed stimulus material in an experimental vignette design and in this way strengthened internal validity. However, there are limitations to the procedure. We collected subjective data from a single source, a method likely to introduce common method bias. Yet, it is difficult to devise of a measure of an individual’s goals and expectations as to how probable their achievement is that would not use self-report. Furthermore, although Aguinis and Bradley (2014) recommend the experimental vignette methodology to better understand individuals’ decision-making processes, especially with regard to work-related behaviors that are not easily observable, there remains a gap between the artificial nature of the situations depicted in the vignettes and real-world circumstances. Describing the situations as realistically as possible helps to increase external validity, but cannot reach the level of non-experimental research. To keep the vignette experiment as simple as possible (Atzmüller and Steiner, 2010), we used only two goals and two results for each vignette. One can imagine that in real life employees consider a greater number of goals and results when deciding about their attendance behavior. Moreover, research has identified a large number of factors influencing attendance behavior. Of these, we systematically extracted relevant and feasible variables. However, of these the eight vignettes represented only a selection. Although we can assume that several of the selected factors were relevant to each participant, they might have mentioned others if asked. In addition, the convenience sample gained via social media was relatively small and not representative of the population.

Thus, further studies should use a design in which participants can state their own goals and outcomes that they would consider when making a decision. Although in terms of the number of independent variables, the sample size was sufficient (Moons et al., 2014; Peduzzi et al., 1996; Pavlou et al., 2015), it would be desirable to obtain a larger sample than the current one for this purpose. That would provide a suitable knowledge base from which occupational health-relevant hypotheses and organizational interventions may be derived to investigate and manage attendance behavior.

## Data Availability Statement

The raw data supporting the conclusions of this article will be made available by the authors, without undue reservation.

## Ethics Statement

Ethical review and approval was not required for the study on human participants in accordance with the local legislation and institutional requirements. The patients/participants provided their written informed consent to participate in this study.

## Author Contributions

Both authors contributed to the study conception and design, performed the material preparation, data collection, and analysis, wrote the manuscript, commented on previous versions of the manuscript, and read and approved the final manuscript.

## Conflict of Interest

The authors declare that the research was conducted in the absence of any commercial or financial relationships that could be construed as a potential conflict of interest.
